# Insulin-Induced Cardiomyocytes Hypertrophy That Is Prevented by Taurine via β-alanine-Sensitive Na^+^-Taurine Symporter

**DOI:** 10.3390/nu13113686

**Published:** 2021-10-20

**Authors:** Ashley Jazzar, Danielle Jacques, Ghassan Bkaily

**Affiliations:** Department of Immunology and Cell Biology, Faculty of Medicine and Health Sciences, Université de Sherbrooke, Sherbrooke, QC J1H 5N4, Canada; ashley.jazzar@usherbrooke.ca (A.J.); danielle.jacques@usherbrooke.ca (D.J.)

**Keywords:** taurine, insulin, cardiomyocytes, hypertrophy, sodium, calcium, cAMP response element binding protein, CREB, β-alanine, Na^+^/Ca^2+^ exchanger, NCX, Na^+^/H^+^ exchanger, NHE1

## Abstract

Although insulin-induced cardiac hypertrophy is reported, very little information is available on the hypertrophic effect of insulin on ventricular cardiomyocytes and the regulation of sodium and calcium homeostasis. Taurine is a non-essential amino acid synthesized by cardiomyocytes and the brain and is present in low quantities in many foods, particularly seafood. The purpose of this study was to investigate whether chronic exposure to insulin induces hypertrophy of ventricular cardiomyocytes that are associated with changes in Na^+^ and Ca^2+^ homeostasis and whether taurine pre-treatment prevents these effects. Our results showed that chronic treatment with insulin leads to cardiomyocyte hypertrophy that is associated with an increase in basal intracellular Na^+^ and Ca^2+^ levels. Furthermore, long-term taurine treatment prevents morphological and ionic remodeling induced by insulin. In addition, blocking the Na^+^-taurine co-transporter prevented the taurine antihypertrophic effect. Finally, the insulin-induced remodeling of cardiomyocytes was associated with a decrease in the ratio of phospho-CREB (pCREB) to total cAMP response element binding protein (CREB); taurine prevented this effect. In conclusion, our results show that insulin induces ventricular cardiomyocyte hypertrophy via downregulation of the pCREB/tCREB level and that chronic taurine treatment prevents this effect.

## 1. Introduction

Two major types of cardiac hypertrophy exist: physiological (observed in the heart of athletes) [1,2] and pathological. The latter is induced by mechanical or chemical factors such as high insulin levels [3]. The effect of insulin on intracellular sodium homeostasis is currently not known. However, similar to the activation of the G protein-coupled receptors (GPCRs), insulin can stimulate calcium influx through the R-type calcium channel, increasing intracellular calcium and inducing intracellular overload of this ion [4,5].

In addition, very few endogenous and exogenous antihypertrophic factors such as taurine were reported to prevent GPCR activation-induced hypertrophy such as angiotensin II [6,7]. Whether this non-essential amino acid also prevents hypertrophy induced by a hypertrophic factor coupled to tyrosine kinase (TKR) receptors such as insulin awaits to be discovered. 

The sodium–taurine co-transporter allows taurine to be co-transported with two Na^+^ ions into the cell, which in the short term, leads to sodium overload followed by a calcium overload via the stimulation of the sodium–calcium exchanger (NCX) [8,9,10,11,12]. A specific antagonist of this transporter is β-alanine [11,13,14]. 

Cyclic adenosine monophosphate (cAMP)-response element-binding protein 1 (CREB) is important for the maintenance of normal physiological cardiac function [15,16,17]. The decrease of this transcriptional factor promotes the development of dilated cardiomyopathy [17]. CREB is considered to be the guardian of the cardiac phenotype [18,19]. Although insulin is known to be a TKR-coupled receptor, it regulates the expression of G-protein alpha-G_i_-2 [20,21] and alpha-G_i_-3 [20]. The activation of these G_i_-proteins will decrease intracellular cAMP, which in turn decreases the activity of CREB. The relation between insulin and CREB needs to be clarified. In addition, whether the antihypertrophic actions of taurine could be mediated via modulation of CREB need to be elucidated. 

We, therefore, studied the effect of insulin on ventricular cardiomyocytes remodeling and whether taurine prevents this remodeling by preventing the effect of insulin on CREB. 

## 2. Materials and Methods

### 2.1. Culture Cells

The method used for the isolation and culture of cardiomyocytes from young adult male rats was previously described [22]. This work was done according to the university’s ethical committee (protocol number 059-16). In summary, the hearts were isolated under aseptic conditions, and the blood was washed out in sterile Ca^2+^- free suspension of minimal essential medium (SMEM) (Sigma, Markham, ON, Canada) solution, which contained 1% penicillin–streptomycin (Thermo Fisher Scientific, Mississauga, ON, Canada). Afterward, the hearts were minced, and the cells were separated by successive trypsinizations and stirring in SMEM (near 22 °C), which contained 0.05% trypsin. Harvested cells were washed by mild centrifugation (200× *g* for 5–10 min). After discarding the supernatant, the cells were resuspended in a fresh culture medium 199 (Thermo Fisher Scientific, Mississauga, ON, Canada) supplemented with 5% fetal bovine serum (FBS). Cell pellets were then diluted with culture medium, plated on a glass coverslip (25 mm) in a petri dish, and placed in an incubator at 37 °C and 5% CO_2_.

For long-term treatment, after two days in culture the cardiomyocytes were treated for 48 h in the absence and presence of different drugs: control, + insulin (80 μU/mL), + insulin (80 μU/mL), and taurine (20 mM), + insulin (80 μU/mL) + taurine (20 mmol), and β-alanine (500 μM) [4,10,23]. The concentrations of the drugs were previously used by our group [4,10,23]. In addition, the concentration of 80 μU/mL was selected as it is similar to the insulin levels observed in hyperglycemia, which is usually between 80–100 μU/mL and were used after testing several concentrations of insulin [4,24]. The concentrations of 20 mM of taurine and 500 µM of β-alanine were selected as they represented the standard concentrations used across the literature and were already published after we tested several concentrations [9,25].

### 2.2. Quantitative 3D Confocal Microscopy

In this study, quantitative three-dimension (3D) confocal microscopy was used [23]. Fluorescent images were obtained using the confocal system MRC1024 (Bio-Rad, Mississauga, ON, Canada) equipped with a Krypton/Argon laser, a UV laser, and a microscope with an inverted epifluorescence (Nikon Eclipse TE2000, Bio-Rad, Mississauga, ON, Canada). Throughout this study, the parameters and conditions of the confocal system were defined as described by Bkaily and his collaborators [26,27]. Once the images were scanned, they were transferred to an O2 Silicon Graphics analysis station equipped with Molecular Dynamics Image Space 3.2 analysis and quantitative reconstruction software (Bio-Rad, Mississauga, ON, Canada). Furthermore, the mean volume values for each compartment (cytosol and nucleus) were measured by isolating the nucleus from the surrounding cytosolic region labeled with Syto-11, which provided 3D information [27]. 

The Fluo-4/AM probe (Molecular Probes, Eugene, OR, USA) was used as a calcium ion indicator and was diluted in Tyrode-BSA from a frozen 1-mM stock in DMSO to a final concentration of 13 μM [26,28]. The same procedure described above was used to load the cells with the sodium-sensitive probe CoroNa green AM and sodium green tetraacetate (molecular probe, Eugene, OR, USA) [26,28]. At the end of each experiment, the nucleus was labeled with the nucleic acid marker Syto-11 (molecular probe) [26,29]. 

### 2.3. DNA, RNA and Protein Extraction

Using the AllPrep DNA/RNA/Proteins Mini Kit (Qiagen, Montreal, QC, Canada), the cells were first lysed and homogenized in RLT buffer. The lysate was then passed through the first AllPrep column. The column was washed, and the DNA was then eluted. Ethanol was added to the liquid that passes through the first AllPrep column, and the liquid was then passed through the second AllPrep “RNeasy” column. The high-quality RNA was then eluted in RNase-free water. APP buffer was added to the liquid that passed through the second “RNeasy” column, and the precipitated proteins were collected by centrifugation. The total intact proteins were dissolved in ALO buffer. The RNA was then eluted with RNase-free water by centrifugation, assayed using a SmartSpecTMPlus spectrometer (Biorad, Mississauga, ON, Canada), and stored at −80 °C for future use. The DNA was then assayed by a Pierce BCA protein assay kit (Thermo Scientific, Waltham, MA, USA) and stored at −20 °C.

### 2.4. Reverse Transcription PCR Analysis 

One µg of RNA was diluted in 10 µL of DEPC water, heated at 75 °C for 5 min, then put on ice for another 5 min. Afterward, 10 µL of a retro-transcription solution containing 4 µL of 5 X AMV RT buffer (Roche Applied Science, Indianapolis, IN, USA), 2 µL of 10 mM dNTPs (Invitrogen, Carlsbad, CA, USA), 2.4 µL of Oligo dT (IDT, San Jose, CA, USA), 0.6 µL of RNase inhibitor (Roche Applied Science, Indianapolis, IN, USA), and 1.5 µL of RT AMV (Roche Applied Science, Indianapolis, IN, USA) was added to the RNA dilution for each sample and incubated for 1 h at 42 °C. To stop the reaction, the enzyme was inactivated at 95 °C for 5 min. 

The primer sequence used for the PNA was sense 5′-GCT GGA CCA TTT GGA AGA AA-3′ and anti-sense 5′-TTG CTT TTT AGG GCA GA-3′. The endogenous gene we used as a control was RPLPO (large ribosomal protein P0). The primers were: sense 5′-GCA ATG TTG CCA GTG TCT G-3′ and antisense 5′-GCC TTG ACC TTT TCA GCA A-3′. To perform a PCR, first, a master reaction was concocted with 15.45 µL of sterile water, 2.0 µL of NEB buffer (New England Biolabs, Pickering, ON, Canada), 0.45 µL of 10 mM dNTP(s) (Invitrogen, Carlsbad, CA, USA), 0.45 µL of each primer (IDT, San Jose, CA, USA), and 0.2 µL of TAQ (New England Biolabs, Pickering, ON, Canada) times the sample number. Next, the master solution was dispensed by 19 µL into each of the PCR tubes, then 1.0 µL of each sample cDNA was added in duplicate. The tubes were then tightly closed, and the final reaction was vortexed. The tubes were placed in the MyCycler PCR (Biorad, Mississauga, ON, Canada) according to the following standard protocol for PNA (1 cycle of 3:00 to 95 °C, 34 cycles of (0:45 to 94 °C, 0:45 to 60 °C, 1:30 to 72 °C), and one cycle of 5:00 to 72 °C) and following that for RPLPO (1 cycle of 3:00 to 95 °C, 30 cycles of (0:45 to 94 °C, 0:45 to 60 °C, 1:30 to 72 °C), and one cycle of 5:00 to 72 °C). At the end of the PCR, the tubes were stored at 4 °C until migration on DNA gel. 

The DNA gel consisted of 1% agarose (USB corporation, Cleveland, OH, USA), TAE 1X (TAE 50X: 242 g Tris (Sigma-Aldrich, St. Louis, MO, USA), 100 mL Na2EDTA pH = 8.0 0.5 M (Sigma-Aldrich, St. Louis, MO, USA), 57.1 mL of glacial acetic acid (Laboratoire MAT, Montreal, QC, Canada), and 2.5 µL of ethidium bromide (EMD Chemicals, Gibbstown, NJ, USA). The PCR products (5 µL) were diluted in sterile water (4 µL) and then in 1X loading buffer (1 µL) (New England Biolabs, Pickering, ON, Canada). They were then loaded into the gel. Migration was performed for approximately 20–30 min at 150 V and 100 mA. The bands obtained from the DNA gel were analyzed by densitometry with the MCID program (InterFocus Imaging Ltd., Linton, Cambridge, UK).

### 2.5. Western Blot

The method used was similar to that previously reported [30,31,32]. Briefly, equivalent amounts of proteins (50 μg) were separated by polyacrylamide gel electrophoresis (10%) and transferred onto a nitrocellulose membrane (Amersham Life Science, Piscataway, NJ, USA). The proteins were then stained with a Ponceau red solution to ensure transfer efficiency. The membranes were incubated for 2 h at room temperature in a blocking solution composed of phosphate buffer solution (PBS) 1X (NaCl 137 mM, 2.7 mM KCl, 10 mM Na_2_HPO_4_, 1.8 mM KH_2_PO_4_, pH 7.4), 5% non-fat dry milk (Carnation; Smucker Foods Canada Inc., Markham, ON, Canada), and 0.05% Tween-20 (Bio-Rad Laboratories, Mississauga, ON, Canada). The membranes were then incubated overnight at 4 °C on a shaker with one of the following primary rabbit-derived antibodies: monoclonal recombinant anti-CREB (phospho S133) antibody [E113] (ab32096; Abcam, Toronto, ON, Canada) or monoclonal recombinant anti-CREB antibody [E306] (ab32515; Abcam, Toronto, ON, Canada). Equal loading of the proteins was confirmed, and the blots were also probed with a mouse monoclonal antibody against β-actin (Abcam, Toronto, ON, Canada). The membranes were then washed three times and incubated with one of the following secondary antibodies: peroxidase-conjugated anti-rabbit IgG monoclonal antibody (Cedarlane, Cytiva, UK). Additionally, negative controls were carried out in the presence of the blocking peptide for each antibody. After three washes, the immune complexes were detected by chemiluminescence (Western Lightning Plus-ECL; Perkin-Elmer, Waltham, MA, USA) and visualized by autoradiography on BIOMAX type film (Kodak, Rochester, NY, USA).

### 2.6. Densitometry

The films were digitized using an Imaging Research Inc. system equipped with an MTI CCD72 camera. The densities of the bands were obtained using the MCID Basic-M5 software (Imaging Research Inc., Catharines, ON, Canada). The density value obtained for each band represents its density multiplied by its surface area, followed by subtracting the background. Then, the ratio of the phosphorated CREB (pCREB) and total CREB (tCREB) band densities to the β-actin band density were calculated. The ratio of pCREB/tCREB was used to compare the density ratio in different experimental conditions.

The Western blot of cardiomyocytes from well plates of different animals was washed with phosphate-buffered saline (PBS), scraped with 150 μL Laemmli buffer containing 2-mercaptoethanol (βME), sonicated, and heated at 56 °C for 10 min. Proteins were separated in a 12% or 15% SDS-PAGE. After transfer, PVDF membranes were probed with anti-tubulin (1/10,000), anti-NPTII (1/4000), anti-flag (1/1000), and anti-HA (1/5000, mouse) antibodies overnight at 4 °C. After three washes, membranes were incubated for 1 h at room temperature with goat anti-rabbit IgG-HRP (1/10,000) or anti-mouse IgG-HRP (1/10,000) antibodies. Proteins were detected with the Western Lightning ECL reagent (#NEL103001EA, Perkin Elmer, Guelph, ON, Canada) according to the manufacturer’s instructions and the ImageQuant LAS 4000 system (# 28955811, GE Healthcare, Mississauga, ON, Canada).

The fluorescence intensity measurements of calcium and sodium are presented as mean intracellular fluorescence intensity values [26,29]. All the values are expressed as standard error of the mean (SEM), where “N” is the number of the adult rats, and “n” is the number of cells [26,29]. Statistical significance was determined using the ANOVA test of repeated measurements for matched values, followed by the Bonferroni multiple comparison tests, where a *p*-value < 0.05 was considered significant [26,29].

## 3. Results

### 3.1. Modulation of the Whole Cell and Nuclear Volumes by a 48-h Treatment with Insulin (80 μU/mL), Taurine (20 mM), and β-alanine (500 μM) of Ventricular Cardiomyocytes

In the first series of experiments, using quantitative 3D confocal microscopy, we studied the effect of a 48-h treatment with insulin (80 μU/mL) on the whole-cell, cytoplasm, and nuclear volume levels

**Figure 1:**
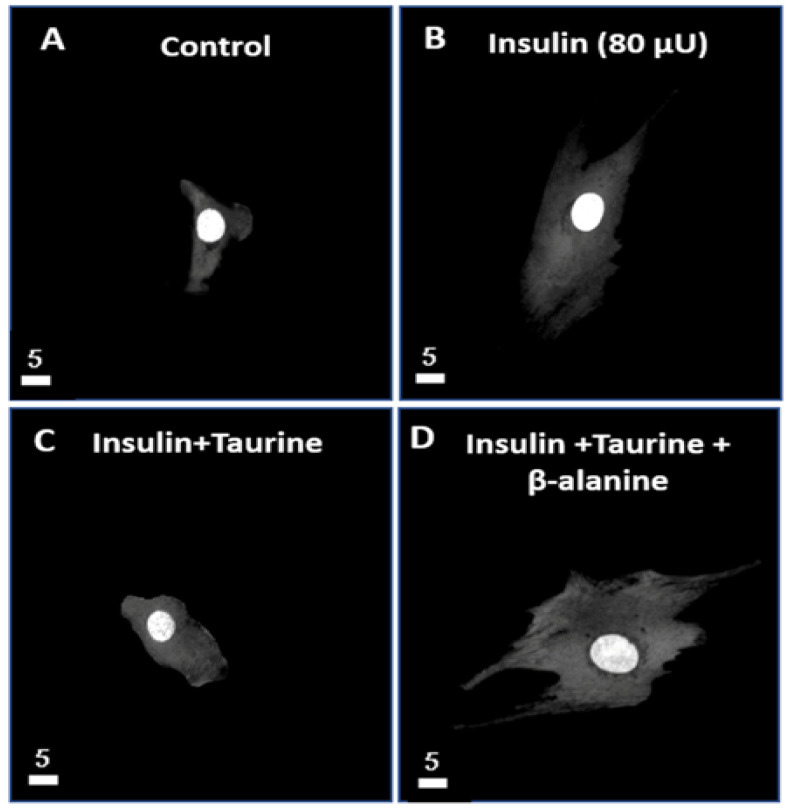
Effect of a 48-h treatment with insulin (80 μU/mL), insulin (80 μU/mL) with taurine (20 mM), and insulin (80 μU/mL) with taurine (20 mM) and β-alanine (500 μM) on the volume of adult rat ventricular cardiomyocytes. Examples of quantitative 3-D images (top view) of isolated ventricular cardiomyocytes in absence of treatment (**A**), in presence of insulin (80 μU/mL) (**B**), in presence of insulin (80 μU/mL) + taurine (20 mM) (**C**), and in presence of insulin (80 μU/mL) + taurine (20 mM) + β-alanine (500 μM) (**D**). The white bar scale is in μm. (**A**–**D**) are different cells.

**Figure 2:**
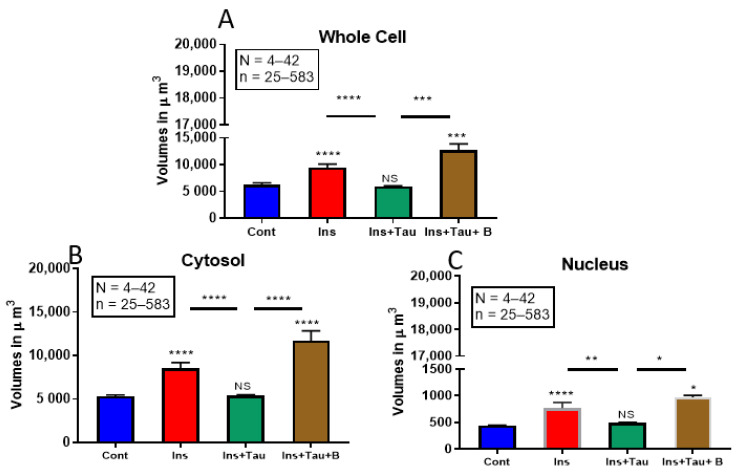
Histograms showing the effect of a 48-h treatment with insulin (80 μU/mL), insulin (80 μU/mL) with taurine (20 mM), and insulin (80 μU/mL) with taurine (20 mM) and β-alanine (500 μM) on the volume of adult rat ventricular cardiomyocytes. The results show the volume of the whole cell (**A**), the cytosol (**B**), and the nucleus (**C**) in the absence of treatment and in the presence of a 48-h treatment of insulin (80 μU/mL), insulin (80 μU/mL) + taurine (20 mM), and insulin (80 μU/mL) + taurine (20 mM) + β-alanine (500 μM). The values are represented as mean ± standard error of the mean. N represents the number of animals, and n represents the number of cells. **** *p* < 0.0001, *** *p* < 0.001, ** *p* < 0.01, and * *p* < 0.05 were considered significant. NS = non-signifiant. N was 42 (control), 42 (insulin), 7 (insulin + taurine), and 4 (insulin + taurine + β-alanine) for the different experiments. n was 226 (control), 158 (insulin), 33 (insulin + taurine), and 57 (insulin + taurine + β-alanine) for the different cells. Cont = control; ins = insulin; tau = taurine; and B = β-alanine. The volume of the cell is in μm^3^.

Figure 1 shows examples, and Figure 2 summarizes the results. As seen in Figure 1A,B, there was an apparent increase in the whole-cell, cytosol, and nuclear volumes after 48 h of treatment with insulin (80 μU/mL) (Figure 1B) when compared to the non-treated cardiomyocytes (Figure 1A). As seen in Figure 2, this increase was highly significant in the whole-cell (Figure 2A *p* < 0.0001), the cytosol (Figure 2B *p* < 0.0001), and the nuclear volumes (Figure 2C *p* < 0.0001) when compared to the non-treated cardiomyocytes. 

In the second series of experiments, we treated the cells with insulin (80 μU) in the presence of taurine (20 mM) for 48 h to verify whether this non-essential amino acid can prevent ventricular cardiomyocytes hypertrophy (Figure 1 and Figure 2) induced by chronic insulin treatment. Figure 1A–C shows examples, and Figure 2 summarizes the results. As shown in Figure 1, taurine prevents the increase in ventricular cardiomyocytes hypertrophy (Figure 1C) induced by insulin (Figure 1B). In addition, there was no significant increase of the whole-cell (Figure 2A), cytosol (Figure 2B), and nuclear volumes (Figure 2C) induced by insulin in the presence of taurine when compared to the non-treated cells (Figure 2). 

In the third series of experiments, we used the potent and specific blocker of the sodium–taurine co-transporter, β-alanine (500 μM) [10,23]. Figure 1 shows examples, and Figure 2 summarizes the results. As seen in Figure 1D, pre-treatment with β-alanine (500 μM) prevented taurine from blocking the cardiomyocyte hypertrophy induced by insulin (Figure 1D). Furthermore, as seen in Figure 2, the presence of β-alanine (500 μM) significantly prevented the antihypertrophic effect of taurine in the whole-cell (Figure 2A *p* < 0.001), the cytosol (Figure 2B *p* < 0.0001), and the nuclear volumes (Figure 2C *p* < 0.05) when compared to the non-treated cardiomyocytes.

**Figure 3:**
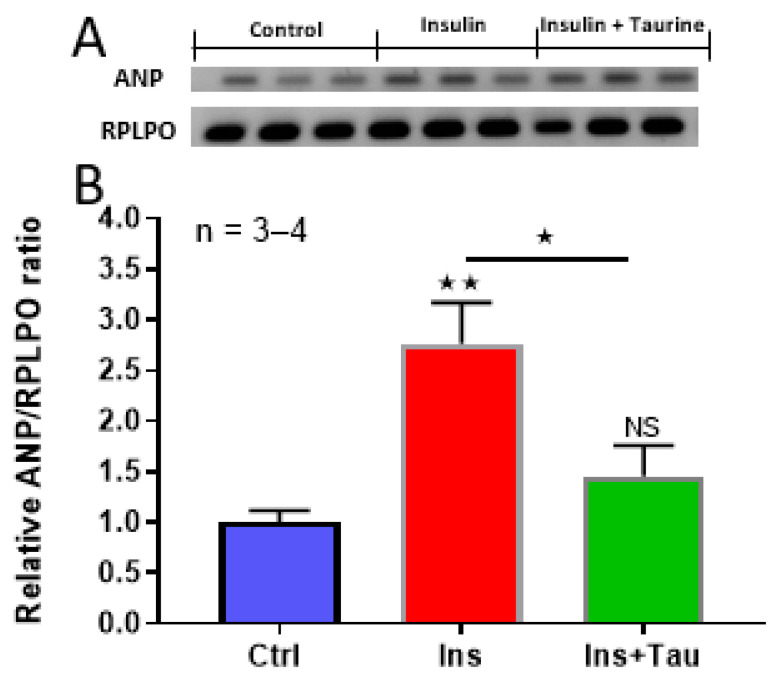
RT-PCR showing the effect of a 48-h treatment with insulin (80 μU/mL) and insulin (80 μU/mL) with taurine (20 mM) on the relative ANP/RPLPO ratio of ventricular cardiomyocytes. (**A**): Autoradiography showing the levels of ANP of ventricular cardiomyocytes in the absence of treatment, treated with insulin (80 μU/mL) and treated with insulin (80 μU/mL) + taurine (20 mM). (**B**): The statistical comparison of the relative ANP/RPLPO ratio was calculated using the densitometric measurements of the corresponding bands. The values are represented as mean ± standard error of the mean. n represents the number of wells. * *p* < 0.05 and ** *p* < 0.01. ANP = atrial natriuretic peptide; RPLPO = large ribosomal protein P0; NS = non-significant.

In the last series of experiments, using RT-PCR, we verified whether the increase in the cell and nuclear volumes was accompanied by the marker of hypertrophy ANP [33,34]. Figure 3 shows examples (A) and summarizes (B) the results. As seen in this figure, insulin significantly increased the ratio of ANP/RPLPO (*p* < 0.001), and taurine completely prevented this effect. 

### 3.2. Modulation of Cytosolic and Nuclear Sodium Levels by Insulin, Insulin + Taurine, and Insulin + Taurine + β-alanine in Ventricular Cardiomyocytes

The purpose of this series of experiments was to verify whether the increase in cell volume by chronic treatment with insulin modulates the resting levels of intracellular sodium and whether the preventive effect of taurine on this hypertrophy was modulated via preventing intracellular sodium overload. 

**Figure 4:**
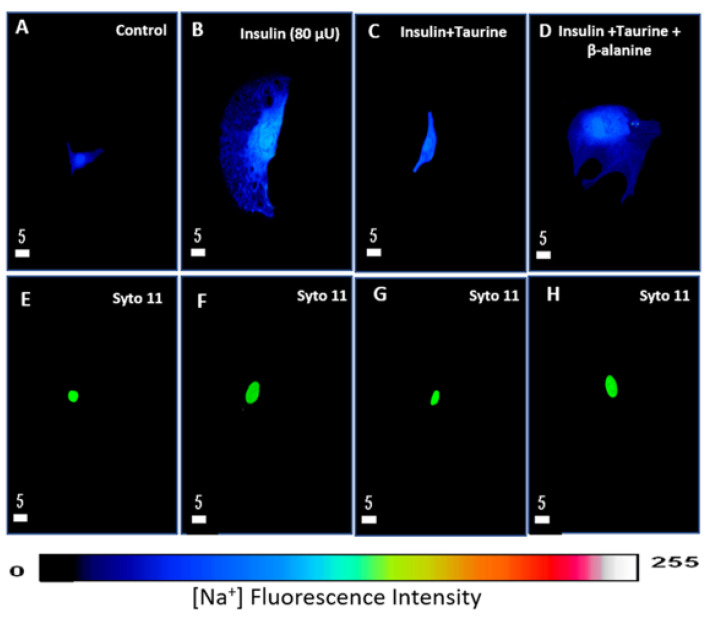
Examples of quantitative 3-D confocal images (top view) showing the distribution and fluorescence intensity of intracellular sodium in cardiomyocytes in the absence of treatment (**A**), in the presence of insulin (80 μU/mL) (**B**), in the presence of insulin (80 μU/mL) + taurine (20 mM) (**C**), and in the presence of insulin (80 μU/mL) + taurine (20 mM) + β-alanine (500 μM) (**D**). Panels (**E**–**H**) represent the Syto-11 labeled nuclei of the cells in panels (**A**–**D**). The pseudo-color bar represents Na^+^ fluorescence intensity ranging from 0 (absence of fluorescence) to 255 (maximum fluorescence). The white scale bar is in μM.

**Figure 5:**
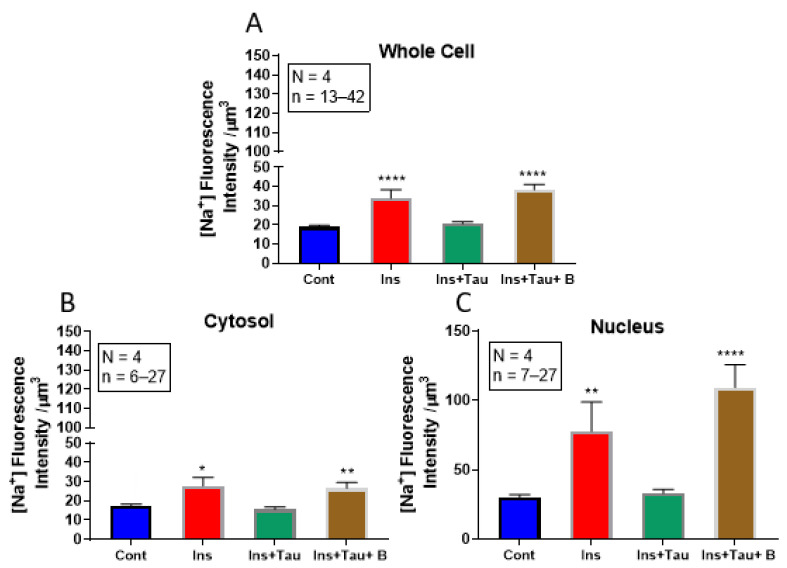
Histograms showing the effect of 48-h treatment with insulin (80 μU/mL), insulin (80 μU/mL) with taurine (20 mM), or insulin (80 μU/mL) with taurine (20 mM) and β-alanine (500 μM) on whole-cell (**A**), cytosolic (**B**), and nuclear (**C**) sodium levels of cardiomyocytes. The values are represented as mean ± standard error of the mean. N represents the number of animals, and n represents the number of cells. * *p* < 0.05, ** *p* < 0.01, and **** *p* < 0.0001. Cont = control; ins = insulin; tau = taurine, and B = β-alanine. Sodium relative concentration is expressed in μm^3^.

In the first series of experiments, after 48 h of treatment with insulin (80 μU/mL), the cells were loaded with sodium fluorescence probes, CoroNa green, and sodium green. Figure 4A,B shows examples, and Figure 5 summarizes the results. As shown in these two figures, there was a significant increase in the whole cell (*p* < 0.0001), as well as in the cytosol (*p* < 0.05) and nucleus (*p* < 0.01) sodium levels that were associated with an apparent increase in the whole cell (Figure 4B) and nuclear volumes (Figure 4F) when compared to non-treated cardiomyocytes (Figure 4A,E). In addition, the nucleus of each cell was labeled with Syto-11 (Figure 4E,F) to locate its position with respect to the whole cell (Figure 4A,B).

Using the same protocol, the cells were treated for 48 h with insulin (80 μU/mL) + taurine (20 mM) in another series of experiments. Figure 4C,G show examples, and Figure 5 summarizes the results. As shown in Figure 4A–C, the prevention of insulin-induced hypertrophy by taurine was accompanied by the prevention of insulin-induced increase in cytosolic and nuclear sodium levels (Figure 4C and Figure 5) compared to the non-treated cells (Figure 4A and Figure 5). However, as shown in Figure 5, there was no significant increase in the intracellular sodium levels in the whole-cell (Figure 5A), cytosol (Figure 5B), and nucleus volumes (Figure 5C). 

In the last series of experiments, using the same protocol, the cells were treated for 48 h with insulin (80 μU/mL) + taurine (20 mM) + β-alanine (500 μM). Figure 4 shows examples, and Figure 5 summarizes the results. As seen in Figure 4, β-alanine’s presence prevented the effect of taurine on the insulin-induced increase in intracellular sodium (Figure 4D) compared to non-treated cells (Figure 4A). In addition, Figure 5 shows the presence of β-alanine significantly prevented taurine from decreasing an insulin-induced increase in whole-cell (Figure 5A), cytosol (Figure 5B), and nucleus (Figure 5C) volumes when compared to non-treated cells.

### 3.3. Modulation of Cytosolic and Nuclear Calcium Levels by Insulin and Taurine in Adult Rat Ventricular Cardiomyocytes

The purpose of this series of experiments was to verify whether the increase in cell volume by chronic treatment with insulin modulates the resting levels of intracellular calcium and whether the preventive effect of taurine on this hypertrophy is modulated via preventing intracellular calcium overload. 

**Figure 6:**
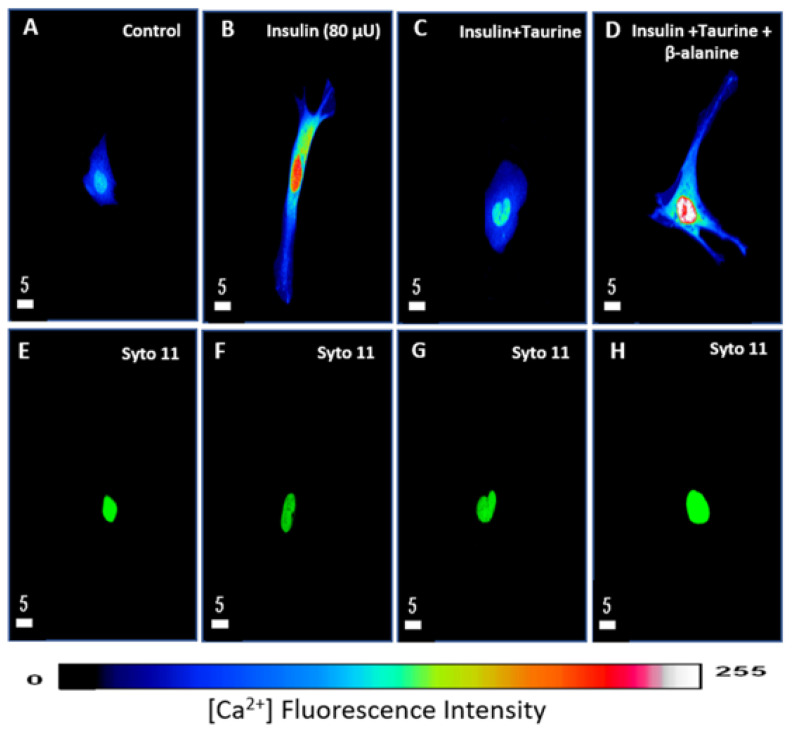
Examples of quantitative 3-D confocal images (top view) showing the distribution and fluorescence intensity of intracellular calcium levels in cardiomyocytes in the absence of treatment (**A**), in the presence of insulin (80 μU/mL) (**B**), in the presence of insulin (80 μU/mL) + taurine (20 mM) (**C**), and in the presence of insulin (80 μU/mL) + taurine (20 mM) + β-alanine (500 μM) (**D**). (**E**–**H**) represent the Syto-11-labeled nuclei of the cells in (**A**–**D**). The pseudo-color bar represents Ca^2+^ fluorescence intensity ranging from 0 (absence of fluorescence) to 255 (maximum fluorescence). The white scale bar is in μM.

**Figure 7:**
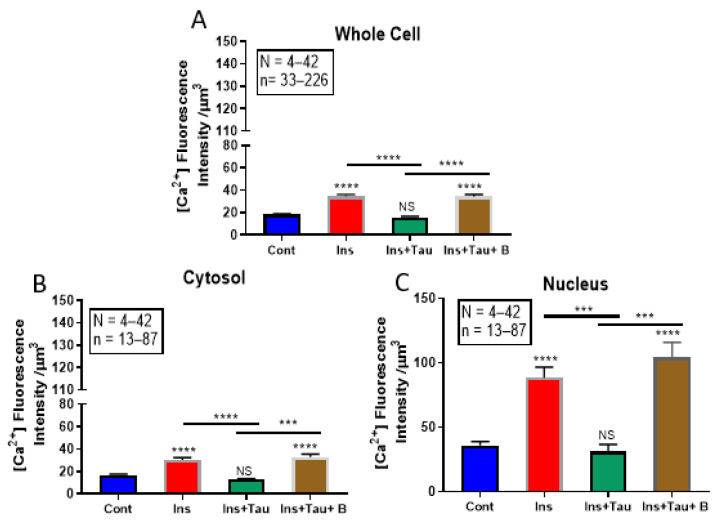
Histograms showing the effect of a 48-h treatment with insulin (80 μU/mL), insulin (80 μU/mL) with taurine (20 mM), and insulin (80 μU/mL) with taurine (20 mM) and β-alanine (500 μM) on the whole-cell (**A**), cytosolic (**B**), and nuclear (**C**) calcium levels of adult rat ventricular cardiomyocytes. The values are represented as mean ± standard error of the mean. N represents the number of animals, and n represents the number of cells., *** *p* < 0.001, and **** *p* < 0.0001. Cont = control; ins = insulin; tau = taurine’ and B = β-alanine. Calcium’s relative concentration is expressed in μm^3^.

In this first series of experiments, we tested the effect of 48 h of treatment with insulin on cytosolic and nuclear calcium. Figure 6 shows examples, and Figure 7 summarizes the results. As seen in Figure 6A,B and Figure 7, there was an apparent increase in the whole-cell and nuclear volumes that was associated with a significant increase in cytosolic (*p* < 0.0001) and nuclear (*p* < 0.0001) free calcium. 

In another series of experiments, using the same protocol, the cells were treated for 48 h with insulin (80 μU/mL) in the presence of taurine (20 mM) to verify whether this non-essential amino acid can prevent intracellular calcium overload induced by chronic insulin treatment. Figure 6 shows examples, and Figure 7 summarizes the results. As seen in Figure 6A–C and Figure 7, taurine completely prevented increased cell volume and cytosolic and nuclear-free calcium induced by insulin (Figure 6C and Figure 7). 

In the third series of experiments, we verified whether the preventive effect of taurine is mediated via its transport through the β-alanine-sensitive sodium–taurine co-transporter. Figure 6 shows examples, and Figure 7 summarizes the results. As seen in these two figures, pre-treatment with β-alanine (500 μM) prevents taurine from inducing a decrease in cytosolic and nuclear calcium caused by long-term treatment with insulin (Figure 6D and Figure 7). 

### 3.4. Effect of Long-Term Treatment with Insulin and Insulin + Taurine on the Ratio of pCREB/tCREB 

**Figure 8:**
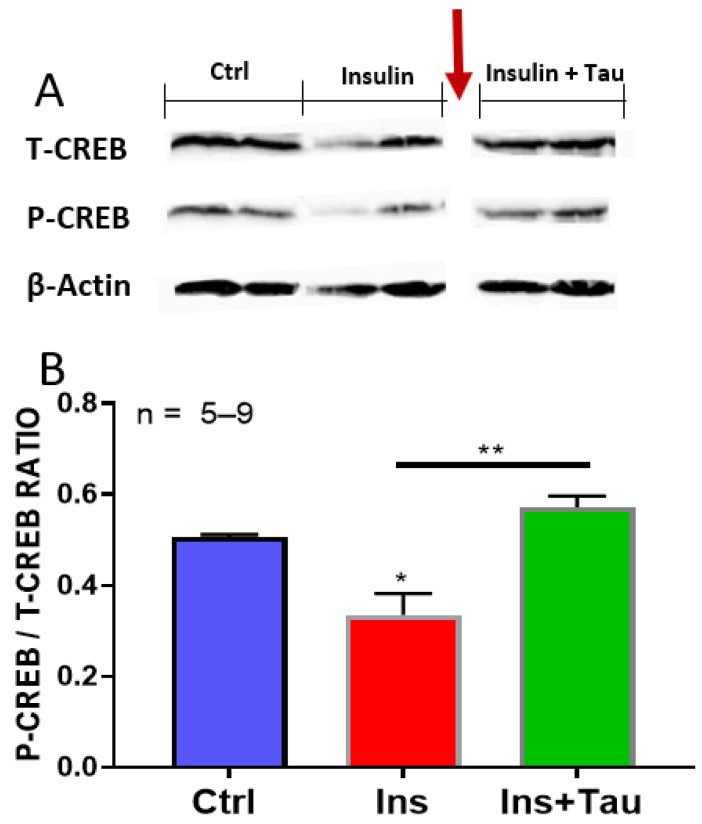
Western blot showing the effect of a 48-h treatment with insulin (80 μU/mL) and insulin (80 μU/mL) with taurine (20 mM) on the pCREB/pCREB ratio. Autoradiography (**A**) showing the ratio level of pCREB/tCREB (**B**) of ventricular cardiomyocytes in absence and presence of treatments, with insulin (80 μU/mL) and with insulin (80 μU/mL) + taurine (20 mM). The band corresponding to the tCREB and pCREB (**A**) were situated approximately at 43 kDA. The band corresponding to the β-actin (**A**) was situated approximately at 42 kDA. The red arrow indicates where the membrane was cut. The values are expressed as mean ± SEM. n is the number of wells. * *p* < 0.05 and ** *p* < 0.01.

Using Western blot, we verified whether insulin-induced morphological remodeling of cardiomyocytes was due to a decrease in the guardian of cell phenotype CREB. As seen in Figure 8, treatment for 48 h with insulin significantly (*p* < 0.05) decreased the ratio of pCREB/tCREB. However, simultaneous treatment with insulin + taurine prevented insulin from inducing a decrease in the ratio of pCREB/tCREB. 

## 4. Discussion

It is accepted that sustained elevation of insulin secretion, such as in hyperglycemia, induces cardiac hypertrophy [35,36,37]. However, this has never been demonstrated in vitro using ventricular cardiomyocytes. Our results showed that 48 h of treatment with insulin did increase cardiomyocytes’ volume. Furthermore, this increase in cell volume was associated with an increase in hypertrophic markers, such as an increase in nuclear volume [33,38] and atrial natriuretic peptide (ANP) levels [33,34]. Thus, our results demonstrated for the first time at the level of ventricular cardiomyocytes that long-term treatment with a relatively high concentration of insulin (80 μU/mL, when compared to fasting concentration, 15–45 μU/mL) induced ventricular cardiomyocytes hypertrophy, which explains its reported hypertrophic effect in in vivo conditions [35,36,37]. 

The increase in cell volume by chronic insulin treatment was accompanied by increased nuclear volume [39,40]. The nuclear volume and the levels of ANP are important markers that permit the distinction between hypertrophy and hyperplasia [39,40,41,42]. Our results showed that the long-term insulin-induced morphological remodeling is associated with a decrease in the ratio of pCREB/tCREB. This decrease is similar to that reported in vascular disease [19], including diabetes [19,43], and may explain at least in part the insulin remodeling of cardiomyocytes morphology found in our experiments. Thus, our results highly suggest that, as in vascular disease [19,43], an insulin-induced decrease in cardiomyocytes CREB levels could be responsible, at least in part, for the development of cardiac hypertrophy in diabetic patients. It is also possible that the decrease in CREB levels by insulin in our experiment could be due to a decrease in protein kinase A (PKA) signaling in cardiomyocytes [44], which decreases the activation of CREB.

Our results also showed that chronic insulin-induced cardiomyocytes hypertrophy was associated with both increases in cytosolic and nuclear-free sodium and calcium. The elevated calcium levels could be due, at least in part, to an increase in calcium influx through the R-type Ca^2+^ channel [45] and/or calcium release from the sarcoplasmic reticulum (SR). It is also possible that the insulin-induced elevation of intracellular sodium would promote calcium influx through the NCX. The increase in intracellular sodium by long-term insulin treatment could be partly due to insulin-induced H^+^ outflux [46], which promotes entry of sodium through the sodium–hydrogen exchanger. The accumulation of intracellular sodium will, in turn, promote calcium influx through the sodium–calcium exchanger. This latter may partly explain the increase in both intracellular sodium and calcium by long-term treatment with insulin. 

Our results showed that treatment with the competitive antagonist of the Na^+^–taurine co-transporter, β-alanine, prevented the antihypertrophic effect of taurine increasing intracellular sodium and calcium levels. These results suggest that the antihypertrophic effect of taurine is due to its influx via its symporter. Furthermore, since the competitive antagonist of the Na^+^–taurine co-transporter prevented the antihypertrophic effect of taurine regarding insulin-induced intracellular sodium overload, this demonstrates that the effect of long-term taurine is due to its modulation of intracellular sodium homeostasis [23]. 

It is reported in the literature that taurine prevents cardiac hypertrophy induced by the protein-coupled receptor agonists [6]. Our results showed for the first time that taurine, similarly to GPCR, is also a pathological agonist of TKR receptors, such as the insulin receptor. These results suggest that the long-term effect of insulin-induced cardiomyocyte hypertrophy may activate the same signaling pathways as those activated by GPCR agonists, such as angiotensin II [47,48]. This should be verified in the future. 

It is also possible that taurine’s antioxidant effect may reduce the level of reactive oxygen species (ROS), which permits better intracellular calcium handling by the mitochondria [49]. This should be verified in the future. 

Furthermore, the fact that the increase in intracellular Na^+^ took place in the presence of the sodium–taurine co-transporter blocker, β-alanine, suggests that the Na^+^–Ca^2+^ exchanger as well as the Na^+^/K^+^ pump both contribute to the evacuation of Na^+^ overload in adult rat cardiomyocytes. This should be verified in the future. 

## 5. Conclusions

In conclusion, our results demonstrated the hypertrophic effect of insulin in ventricular cardiomyocytes. Hence, when a sustained elevation of insulin secretion occurs, such as in the case of hyperglycemia, chronic insulin levels will induce cardiomyocytes hypertrophy, which will promote cardiac hypertrophy [35,36,37]. This will most likely induce an intracellular sodium overload via increasing Na+ influx through NHE1 (sodium-hydrogen exchanger isoform-1). This is followed by an increase in intracellular calcium via an elevation of Ca^2+^ influx through the NCX [50]. Studying the contribution of PKA to CREB activation will not only affect the activation of CREB since the catalytic subunit of PKA will phosphorylate many proteins that could be implicated in the development of cardiac hypertrophy. Among these proteins, all the calcium-dependent signaling, such as phosphorylation of the L-type calcium channel, increases its probability of opening and increases calcium influx through this type of channel [51,52,53]. Furthermore, the catalytic subunit of PKA will also regulate the activity of the potassium channels [54] and other mechanisms [55]. Increased intracellular calcium will activate several signaling and transcriptional factors and genes implicated in cardiomyocyte hypertrophy [11]. In addition, blocking the cAMP pathway is not the only pathway mechanism that activates CREB since they are also cAMP-independent CREB [56].

Finally our results showed that taurine could be used as a cardiac antihypertrophic agent in diabetic and obese patients.

## Data Availability

The data presented in this study are available on request from the corresponding author.

## References

[1] Hunter J.J., Chien K.R. (1999). Signaling Pathways for Cardiac Hypertrophy and Failure. N. Engl. J. Med..

[2] Lemmens K., Doggen K., De Keulenaer G.W. (2007). Role of Neuregulin-1/ErbB Signaling in Cardiovascular Physiology and Disease: Implications for Therapy of Heart Failure. Circulation.

[3] Samak M., Fatullayev J., Sabashnikov A., Zeriouh M., Schmack B., Farag M., Popov A.-F., Dohmen P.M., Choi Y.-H., Wahlers T. (2016). Cardiac Hypertrophy: An Introduction to Molecular and Cellular Basis. Med. Sci. Monit. Basic Res..

[4] Bkaily G., Econornos D., Potvin L., Ardilouze J.-L., Marriott C., Corcos J., Bonneau D., Fong C.N. (1992). Blockade of Insulin Sensitive Steady-State R-Type Ca2+ Channel by PN 200-110 in Heart and Vascular Smooth Muscle. Mol. Cell. Biochem..

[5] Bkaily G., Naik R., Jaalouk D., Jacques D., Economos D., D’Orléans-Juste P., Pothier P. (1998). Endothelin-1 and Insulin Activate the Steady-State Voltage Dependent R-Type Ca2+ Channel in Aortic Smooth Muscle Cells via a Pertussis Toxin and Cholera Toxin Sensitive G-Protein. Mol. Cell. Biochem..

[6] Azuma M., Takahashi K., Fukuda T., Ohyabu Y., Yamamoto I., Kim S., Iwao H., Schaffer S.W., Azuma J. (2000). Taurine Attenuates Hypertrophy Induced by Angiotensin II in Cultured Neonatal Rat Cardiac Myocytes. Eur. J. Pharmacol..

[7] Singh P., Marcu K.B., Goldring M.B., Otero M. (2019). Phenotypic Instability of Chondrocytes in Osteoarthritis: On a Path to Hypertrophy. Ann. N. Y. Acad. Sci..

[8] Bkaily G., Haddad G., Jaalouk D., Gros-Louis N., Benchekroun M.T., Naik R., Pothier P., D’Orléans-Juste P., Bui M., Wang S. (1996). Modulation of Ca2+ and Na+ Transport by Taurine in Heart and Vascular Smooth Muscle. Adv. Exp. Med. Biol..

[9] Bkaily G., Jaalouk D., Haddad G., Gros-Louis N., Simaan M., Naik R., Pothier P. (1997). Modulation of Cytosolic and Nuclear Ca2+ and Na+ Transport by Taurine in Heart Cells. Mol. Cell. Biochem..

[10] Bkaily G., Jaalouk D., Sader S., Shbaklo H., Pothier P., Jacques D., D’Orléans-Juste P., Cragoe E.J., Bose R. (1998). Taurine Indirectly Increases [Ca]i by Inducing Ca2+ Influx through the Na(+)-Ca2+ Exchanger. Mol. Cell. Biochem..

[11] Bkaily G., Simon Y., Jazzar A., Najibeddine H., Normand A., Jacques D. (2021). High Na+ Salt Diet and Remodeling of Vascular Smooth Muscle and Endothelial Cells. Biomedicines.

[12] Hamali B., Pichler S., Wischnitzki E., Schicker K., Burger M., Holy M., Jaentsch K., Molin M., Sehr E.M., Kudlacek O. (2018). Identification and Characterization of the Fasciola Hepatica Sodium- and Chloride-Dependent Taurine Transporter. PLoS Negl. Trop. Dis..

[13] Pasantes-Morales H., Quesada O., Cárabez A., Huxtable R.J. (1983). Effects of the Taurine Transport Antagonist, Guanidinoethane Sulfonate, and β-Alanine on the Morphology of Rat Retina. J. Neurosci. Res..

[14] Dantzler W.H., Silbernagl S. (1976). Renal Tubular Reabsorption of Taurine, Gamma-Aminobutyric Acid (GABA) and Beta-Alanine Studied by Continuous Microperfusion. Pflügers Archiv.

[15] Steven A., Friedrich M., Jank P., Heimer N., Budczies J., Denkert C., Seliger B. (2020). What Turns CREB on? And off? And Why Does It Matter?. Cell. Mol. Life Sci..

[16] Truong V., Anand-Srivastava M.B., Srivastava A.K. (2021). Role of Cyclic AMP Response Element Binding Protein (CREB) in Angiotensin II-Induced Responses in Vascular Smooth Muscle Cells. Can. J. Physiol. Pharmacol..

[17] Watson P.A., Reusch J.E.B., McCune S.A., Leinwand L.A., Luckey S.W., Konhilas J.P., Brown D.A., Chicco A.J., Sparagna G.C., Long C.S. (2007). Restoration of CREB Function Is Linked to Completion and Stabilization of Adaptive Cardiac Hypertrophy in Response to Exercise. Am. J. Physiol. Heart Circ. Physiol..

[18] Kudryavtseva O., Aalkjaer C., Matchkov V.V. (2013). Vascular Smooth Muscle Cell Phenotype Is Defined by Ca2+-Dependent Transcription Factors. FEBS J..

[19] Schauer I.E., Knaub L.A., Lloyd M., Watson P.A., Gliwa C., Lewis K.E., Chait A., Klemm D.J., Gunter J.M., Bouchard R. (2010). CREB Downregulation in Vascular Disease: A Common Response to Cardiovascular Risk. Arterioscler. Thromb. Vasc. Biol..

[20] Livingstone C., MacDonald C., Willett B., Houslay M.D. (1994). Analysis of the Adenylate Cyclase Signalling System, and Alterations Induced by Culture with Insulin, in a Novel SV40-DNA-Immortalized Hepatocyte Cell Line (P9 Cells). Biochem. J..

[21] Moxham C.M., Malbon C.C. (1996). Insulin Action Impaired by Deficiency of the G-Protein Subunit Giα2. Nature.

[22] Bkaily G., Sperelakis N., Doane J. (1984). A New Method for Preparation of Isolated Single Adult Myocytes. Am. J. Physiol..

[23] Bkaily G., Jazzar A., Normand A., Simon Y., Al-Khoury J., Jacques D. (2020). Taurine and Cardiac Disease: State of the Art and Perspectives. Can. J. Physiol. Pharmacol..

[24] Kahn S.E. (2003). The Relative Contributions of Insulin Resistance and Beta-Cell Dysfunction to the Pathophysiology of Type 2 Diabetes. Diabetologia.

[25] Xu Y.-J., Arneja A.S., Tappia P.S., Dhalla N.S. (2008). The Potential Health Benefits of Taurine in Cardiovascular Disease. Exp. Clin. Cardiol..

[26] Bkaily G., Pothier P., D’Orléans-Juste P., Simaan M., Jacques D., Jaalouk D., Belzile F., Hassan G., Boutin C., Haddad G. (1997). The Use of Confocal Microscopy in the Investigation of Cell Structure and Function in the Heart, Vascular Endothelium and Smooth Muscle Cells. Mol. Cell. Biochem..

[27] Bkaily G., Al-Khoury J., Simon Y., Jacques D. (2017). Intracellular Free Calcium Measurement Using Confocal Imaging. Methods Mol. Biol..

[28] Bkaily G., Jacques D., Pothier P. (1999). Use of Confocal Microscopy to Investigate Cell Structure and Function. Methods Enzymol..

[29] Jacques D., Sader S., Perreault C., Fournier A., Pelletier G., Beck-Sickinger A.G., Descorbeth M. (2003). Presence of Neuropeptide Y and the Y1 Receptor in the Plasma Membrane and Nuclear Envelope of Human Endocardial Endothelial Cells: Modulation of Intracellular Calcium. Can. J. Physiol. Pharmacol..

[30] Bkaily G., Sleiman S., Stephan J., Asselin C., Choufani S., Kamal M., Jacques D., Gobeil F., D’Orléans-Juste P. (2003). Angiotensin II AT1 Receptor Internalization, Translocation and de Novo Synthesis Modulate Cytosolic and Nuclear Calcium in Human Vascular Smooth Muscle Cells. Can. J. Physiol. Pharmacol..

[31] Bkaily G., Chahine M., Al-Khoury J., Avedanian L., Beier N., Scholz W., Jacques D. (2015). Na(+)-H(+) Exchanger Inhibitor Prevents Early Death in Hereditary Cardiomyopathy. Can. J. Physiol. Pharmacol..

[32] Chahine M., Bkaily G., Nader M., Al-Khoury J., Jacques D., Beier N., Scholz W. (2005). NHE-1-Dependent Intracellular Sodium Overload in Hypertrophic Hereditary Cardiomyopathy: Prevention by NHE-1 Inhibitor. J. Mol. Cell. Cardiol..

[33] Bkaily G., Abou Abdallah N., Simon Y., Jazzar A., Jacques D. (2020). Vascular Smooth Muscle Remodeling in Health and Disease. Can. J. Physiol. Pharmacol..

[34] Gardner D.G. (2003). Natriuretic Peptides: Markers or Modulators of Cardiac Hypertrophy?. Trends Endocrinol. Metab..

[35] Galderisi M., Anderson K.M., Wilson P.W., Levy D. (1991). Echocardiographic Evidence for the Existence of a Distinct Diabetic Cardiomyopathy (the Framingham Heart Study). Am. J. Cardiol..

[36] Grossman E., Shemesh J., Shamiss A., Thaler M., Carroll J., Rosenthal T. (1992). Left Ventricular Mass in Diabetes-Hypertension. Arch. Intern. Med..

[37] Lin X., Yang P., Reece E.A., Yang P. (2017). Pregestational Type 2 Diabetes Mellitus Induces Cardiac Hypertrophy in the Murine Embryo through Cardiac Remodeling and Fibrosis. Am. J. Obstet. Gynecol..

[38] Sadoshima J., Izumo S. (1997). The Cellular and Molecular Response of Cardiac Myocytes to Mechanical Stress. Annu. Rev. Physiol..

[39] Gerdes A.M., Liu Z., Zimmer H.G. (1994). Changes in Nuclear Size of Cardiac Myocytes during the Development and Progression of Hypertrophy in Rats. Cardioscience.

[40] Jacques D., Bkaily G. (2019). Endocardial Endothelial Cell Hypertrophy Takes Place during the Development of Hereditary Cardiomyopathy. Mol. Cell. Biochem..

[41] Komi P. (2002). Strength and Power in Sport: Olympic Encyclopedia of Sports Medicine.

[42] Ulrich-Lai Y.M., Figueiredo H.F., Ostrander M.M., Choi D.C., Engeland W.C., Herman J.P. (2006). Chronic Stress Induces Adrenal Hyperplasia and Hypertrophy in a Subregion-Specific Manner. Am. J. Physiol. Endocrinol. Metab..

[43] Watson P.A., Nesterova A., Burant C.F., Klemm D.J., Reusch J.E. (2001). Diabetes-Related Changes in CAMP Response Element-Binding Protein Content Enhance Smooth Muscle Cell Proliferation and Migration. J. Biol. Chem..

[44] Eyster C.A., Matsuzaki S., Newhardt M.F., Giorgione J.R., Humphries K.M. (2020). Diabetes Induced Decreases in PKA Signaling in Cardiomyocytes: The Role of Insulin. PLoS ONE.

[45] Bkaily G., Avedanian L., Al-Khoury J., Chamoun M., Semaan R., Jubinville-Leblanc C., D’Orléans-Juste P., Jacques D. (2015). Nuclear Membrane R-Type Calcium Channels Mediate Cytosolic ET-1-Induced Increase of Nuclear Calcium in Human Vascular Smooth Muscle Cells. Can. J. Physiol. Pharmacol..

[46] Goguen J.M., Halperin M.L. (1993). Can Insulin Administration Cause an Acute Metabolic Acidosis in Vivo? An Experimental Study in Dogs. Diabetologia.

[47] Huxtable R.J. (1992). Physiological Actions of Taurine. Physiol. Rev..

[48] Khac L.D., Naze S., Harbon S. (1994). Endothelin Receptor Type A Signals Both the Accumulation of Inositol Phosphates and the Inhibition of Cyclic AMP Generation in Rat Myometrium: Stimulation and Desensitization. Mol. Pharmacol..

[49] Birben E., Sahiner U.M., Sackesen C., Erzurum S., Kalayci O. (2012). Oxidative Stress and Antioxidant Defense. World Allergy Organ. J..

[50] Karmazyn M., Kilić A., Javadov S. (2008). The Role of NHE-1 in Myocardial Hypertrophy and Remodelling. J. Mol. Cell. Cardiol..

[51] Bkaily G., Sperelakis N. (1984). Injection of protein kinase inhibitor into cultured heart cells blocks calcium slow channels. Am. J. Physiol..

[52] Smith R.D., Goldin A.L. (1992). Protein kinase A phosphorylation enhances sodium channel currents in Xenopus oocytes. Am. J. Physiol..

[53] Johnson B.D., Scheuer T., Catterall W.D. (1994). Voltage-dependent potentiation of L-type Ca2+ channels in skeletal muscle cells requires anchored cAMP-dependent protein kinase. Proc. Natl. Acad. Sci. USA.

[54] Wong R., Schlichter L.C. (2014). PKA reduces the rat and human KCa3.1 current, CaM binding, and Ca2+ signaling, which requires Ser332/334 in the CaM-binding C terminus. J. Neurosci..

[55] Liu Y., Chen J., Xia P., Stratakis C.A., Chen Z. (2021). Loss of PKA regulatory subunit 1α aggravates cardiomyocyte necrosis and myocardial ischemia/reperfusion injury. J. Biol. Chem..

[56] Carriba P., Pardo L., Parradamas A., Lichtenstein M.P., Saura C.A., Pujol A., Masgrau R., Galea E. (2012). ATP and noradrenaline activate CREB in astrocytes via noncanonical Ca(2+) and cyclic AMP independent pathways. GLIA.

